# ﻿A new species of *Nidirana* (Anura, Ranidae) from northern Guangxi, China

**DOI:** 10.3897/zookeys.1135.94371

**Published:** 2022-12-12

**Authors:** Wei-Cai Chen, Jian-Ping Ye, Wan-Xiao Peng, Peng Li, Tong-Ping Su, Gui-Dong Yu, Zhi-Ying Cheng

**Affiliations:** 1 Key Laboratory of Environment Change and Resources Use in Beibu Gulf Ministry of Education, Nanning Normal University, Nanning 530001, China; 2 Guangxi Key Laboratory of Earth Surface Processes and Intelligent Simulation, Nanning Normal University, Nanning 530001, China; 3 Maoershan National Nature Reserve, Guilin 541000, China; 4 Guangxi Forest Inventory and Planning Institute, Nanning 530001, China

**Keywords:** Bioacoustics, morphology, nest construction, phylogeny

## Abstract

A new species of music frog, *Nidiranaguibeiensis***sp. nov.**, is described from northern Guangxi, China. Based on two mtDNA fragments analyzed, phylogenetic trees reveal that *N.guibeiensis***sp. nov.** is most closely related to *N.leishanensis*. However, the new species can be identified by conspicuous diagnostic morphological characteristics as well as bioacoustics. In contrast to the known *Nidirana* species, the advertisement calls of the new species can be divided into three types, calls with one, two, and three notes. In addition, the new species has nest construction behavior, which is inconsistent with *N.leishanensis*. *Nidiranaguibeiensis***sp. nov.** occurs in paddy fields or still pools at 300–1300 m a.s.l.

## ﻿Introduction

The genus *Nidirana* Dubois, 1992 is widespread in eastern and southeastern Asia (Frost 2021; AmphibiaChina 2022). Recently, the known diversity of *Nidirana* has increased dramatically, due to combined morphological, molecular, and bioacoustical analyses (Li et al. 2019; Lyu et al. 2020a, 2020b, 2021). To date, there are 17 recognized *Nidirana* species. Most have been reported in the past five years (AmphibiaChina 2022). Five *Nidirana* species have been confirmed to occur in Guangxi: *N.guangxiensis* Mo, Lyu, Huang, Liao & Wang, 2021, *N.leishanensis* Li, Wei, Xu, Cui, Fei, Jiang, Liu & Wang, 2019, *N.shiwandashanensis* Chen, Peng, Li & Liu, 2022, *N.xiangica* Lyu & Wang, 2020a, and *N.yaoica* Lyu, Mo, Wan, Li, Pang & Wang, 2019. *Nidiranaadenopleura* (Boulenger, 1909) has always been considered to occur throughout Guangxi (Fei et al. 2009; Mo et al. 2014). However, recent research has indicated the misidentifications of specimens allocated to *N.adenopleura*, and no evidence supports the occurrence of *N.adenopleura* in Guangxi (Lyu et al. 2021; Chen et al. 2022).

In 2022, we conducted surveys in northern Guangxi and collected 15 *Nidirana* specimens. These specimens differ from the known *Nidirana* species in morphology, phylogeny, and bioacoustics. Herein, we describe these specimens as a new species of *Nidirana*.

## ﻿Materials and methods

### ﻿Sampling and morphological examination

**Figure 1.**
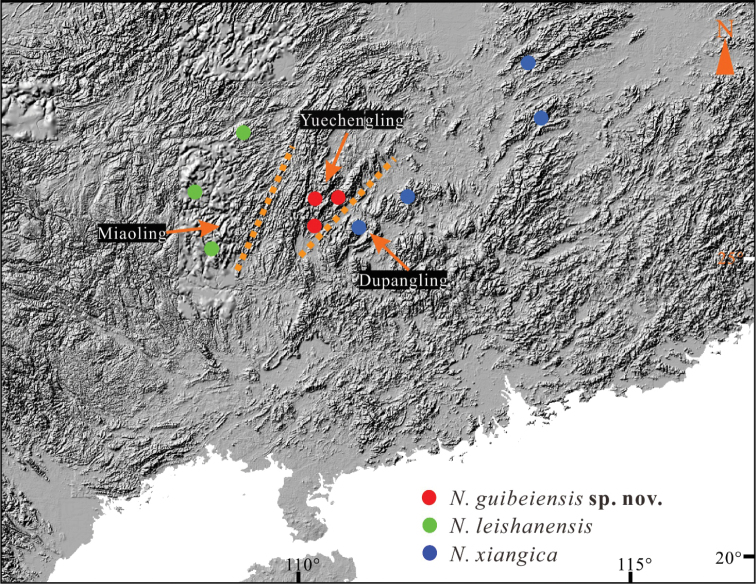
Localities of the new species and its sister taxa. The source of the map came from WorldClim (http://www.worldclim.com/version2).

Fourteen adults and one subadult were collected at the Maoershan National Nature Reserve (*n* = 3), Zhongfeng Town, Ziyuan County (*n* = 11), and Lingtan Town, Xing’an County (*n* = 1) in northern Guangxi, China (Fig. 1). After euthanasia with isoflurane, all specimens were fixed in 10% formalin, then transferred to 75% ethanol, and finally deposited at Nanning Normal University (NNU). Before being fixed, muscle samples were taken and stored in 100% ethanol for molecular analysis. The definition of morphological characteristics and measurements followed Chen et al. (2022). The following measurements were taken with digital calipers to the nearest 0.1 mm:

**SVL** snout-vent length (from the tip of snout to posterior margin of vent);

**HDL** head length (from the tip of snout to the articulation of jaw);

**HDW** head width (head width at the commissure of jaws);

**SNT** snout length (from the tip of snout to the anterior corner of eye);

**IND** internasal distance (distance between nares);

**IOD** interorbital distance (minimum distance between upper eyelids);

**ED** eye diameter (from the anterior corner of eye to posterior corner of eye);

**TD** tympanum diameter (horizontal diameter of tympanum);

**HND** hand length (from the proximal border of the outer palmar tubercle to the tip of Finger III);

**FTL** foot length (from the distal end of the shank to the tip of Toe IV);

**TIB** tibial length (from the outer surface of the flexed knee to the heel).

Sex was identified by examining the nuptial pad and suprabrachial gland. The webbing formula followed Savage (1975).

Geographically and phylogenetically, the new species, *N.leishanensis*, and *N.xiangica* are close to each other. Fourteen adults were measured for comparison, whereas the morphological data of *N.leishanensis* and *N.xiangica* came from the references (Li et al. 2019; Lyu et al. 2020a). A principal component analysis (PCA) and the Mann-Whitney *U* tests were performed on SPSS, based on the adult male specimens. To reduce the impact of allometry, the correct value from the ratio of each character to SVL was calculated, then log-transformed for analyzing. The significance level was set at 0.05. Morphological comparison data came from the collected specimens (Appendix 1) and the references in Table 1.

**Table 1.** Diagnostic characters separating *Nidiranaguibeiensis* sp. nov. from all congeners. Labial tooth row formula from Dubois (1995).

ID	Species	SVL of males (mm)	SVL of females (mm)	Fingers tips	Lateroventral groove on fingers	Relative length of fingers	Toes tips	Lateroventral groove on toes	Tibio-tarsal articulation	Subgular vocal sacs	Nuptial pad	Spinules on dorsal skin	Nest construction	Tadpole labial tooth row formula	Calling	References
1	*N.guibeiensis* sp. nov.	50.2–63.6	54.6	Dilated	Present except finger I	II < IV < I < III	Dilated	Present	Eye-snout	Present	One on finger I	Absent	Present	?	1–3 notes	This study
2	*N.adenopleura*	43.1–57.6	47.6–60.7	Dilated	Present except finger I	II < I < IV < III	Dilated	Present	Snout tip or eye-snout	Present	One on finger I	Entire or posterior	Absent	I:1+1/1+1:II or I:0+0/1+1:I	2–5 regular notes	Lyu et al. (2017, 2020b)
3	*N.chapaensis*	35.5–42.5	41.0–51.8	Dilated	Present except finger I	II < I = IV < III	Dilated	Present	Nostril	Present	Two on finger I	Absent or few above vent	Present	I:1+2/1+1:II	3 notes	Chuaynkern et al. (2010)
4	*N.daunchina*	40.6–51.0	44.0–53.0	Dilated	Absent or rarely present	II < I < IV < III	Dilated	Present	Nostril	Present	One on finger I	Absent	Present	I:1+1/1+1:II or I:1+1/2+2:I	2–5 notes containing a specific first note	Liu (1950); Lyu et al. (2017)
5	*N.guangdongensis*	50.0–58.4	55.3–59.3	Dilated	Present except finger I	II < I < IV < III	Dilated	Present	Nostril	Present	One on finger I	Entire	Absent	?	2–4 regular notes	Lyu et al. (2020a)
6	*N.guangxiensis*	40.2–47.6	49.9–51.0	Dilated	Present on fingers III and IV	II < I < IV < III	Dilated	Present	Nostril	Present	One on finger I	Absent	Present	I: 1+1/1+1:II	6–11 rapidly repeated regular notes	Lyu et al. (2021)
7	*N.hainanensis*	32.8–44.4	?	Dilated	Present	II < I < IV < III	Dilated	Present	Nostril	Present	Absent	Absent	Present	?	2–4 fast-repeated double-notes	Fei et al. (2007, 2009)
8	*N.leishanensis*	49.5–56.4	43.7–55.3	Dilated	Present	II < IV < I < III	Dilated	Present	Eye-snout	Present	Two on fingers I and II	Absent	Absent	I:1+2/ 1+1:II	1 single note	Li et al. (2019)
9	*N.lini*	44.1–63.1	57.7–68.6	Dilated	Present except finger I	II < I < IV < III	Dilated	Present	Beyond snout	Present	One on finger I	Posterior	Absent	I:1+1/1+1:II	5–7 notes containing a specific first note	Chou (1999); Lyu et al. (2017)
10	*N.mangveni*	53.6–59.7	59.7–65.1	Dilated	Present on fingers III and IV	I < II < IV < III	Dilated	Present	Anterior corner of eye	Present	One on finger I	Entire or posterior	Absent	?	2–7 regular notes	Lyu et al. (2020a)
11	*N.nankunensis*	33.3–37.1	37.8–39.5	Dilated	Present except finger I	II < I < IV < III	Dilated	Present	Nostril	Present	One on finger I	Absent or few above vent	Present	I:1+1/1+1:II	13–15 notes containing a specific first note	Lyu et al. (2017)
12	*N.occidentalis*	44.5–53.0	55.6–61.3	Not dilated	Absent	II < I < IV < III	Not dilated	Absent	Eye	Present	One on finger I	Posterior	Absent	?	3–5 regular notes	Lyu et al. (2020b)
13	*N.okinavana*	35.5–42.8	44.6–48.8	Dilated	Present except finger I	II < I < IV < III	Dilated	Present	Eye center-near nostril	Absent	Poorly one on finger I	Absent	Present	I:1+1/1+1:II	10–25 fast-repeated notes	Chuaynkern et al. (2010); Lyu et al. (2017)
14	*N.pleuraden*	46.2–52.3	46.9–61.7	Not dilated	Absent	II < I < IV < III	Not dilated	Absent	Nostril	Present	One on finger I	Posterior	Absent	I:1+1/1+1: II	1–4 regular notes	Lyu et al. (2017, 2020b)
15	*N.shiwandashanensis*	46.2–50.8	48.3	Dilated	Present	II < IV < I < III	Dilated	Present	Eye	Present	One on finger I	Absent	?	I: 1+1/1+1:II	6–8 double-notes	Chen et al. (2022)
16	*N.xiangica*	56.3–62.3	53.5–62.6	Dilated	Present	II < I < IV < III	Dilated	Present	Eye-snout	Present	One on finger I	Entire	Absent	?	2–3 notes containing a specific first note	Lyu et al. (2020a)
17	*N.yaoica*	42.1–45.6	?	Dilated	Present	II < I < IV < III	Dilated	Present	Nostril	Present	One on finger I	Absent	? (Probably present)	?	1–3 fast-repeated regular notes	Lyu et al. (2019)
18	*N.yeae*	41.2–43.5	44.7	Dilated	Absent	II < IV < I < III	Dilated	Present	Eye	Present	One on finger I	Absent	? (Probably absent)	I: 1+1/1+1:II	2–6 notes containing a specific first note	Wei et al. (2020)

### ﻿Phylogenetic analyses

Bayesian inference (BI) and maximum likelihood (ML) methods were used to analyze phylogenetical relationships based on partial 16S ribosomal RNA gene (16S, ~1050 bp) and partial cytochrome oxidase subunit I gene (COI, ~640 bp) sequences analyses. The two mtDNA fragments were amplified and sequenced following Lyu et al. (2019). PCR reaction conditions included 94 °C for 5 min; 35 cycles of denaturing at 94 °C for 35 s, annealing at 55 °C (for 16S)/52 °C (for COI) for 45 s, and extending at 72 °C for 60 s. Sequences were sequenced using an ABI3730 automated DNA sequencer in Sangon Biotech (Shanghai) Co., Ltd (Guangzhou, China). In addition, homologous sequences of *Nidirana* species downloaded from GenBank were included in our phylogenetic analysis (Table 2). These sequences contain all holotypes or paratypes of *Nidirana* species known in China. The BI analysis was implemented using MRBAYES v. 3.1.2 (Ronquist and Huelsenbeck 2003). The best-fit model (GTR+I+G) was chosen using JMODELTEST v. 2.1.2 (Posada 2008) based on Akaike and Bayesian information criteria. Two independent runs with four Markov Chain Monte Carlo simulations were performed for 30 million iterations and sampled every 1000^th^ iteration. The first 25% of samples were discarded as burn-in. ML was analyzed on the CIPRES science gateway with 100 rapid bootstrap replicates (Miller et al. 2010) (https://www.phylo.org/portal2). Outgroups follow Chen et al. (2022).

**Table 2.** Information for samples used in phylogenetic analyses in this study. Type locality indicated by an asterisk (*). NNU represents Nanning Normal University; SYS, Sun Yat-sen University; MNHN, Muséum National d’Histoire Naturelle, Paris; NHMG, Natural History Museum of Guangxi; CIB, Chengdu Institute of Biology, Chinese Academy of Sciences.

ID	Species	Locality	Voucher no.	16S	COI	References
1	*N.guibeiensis* sp. nov.	China: Guangxi: Xing’an: Maoershan (paratype)	NNU 00917	ON985180	ON968962	This study
2	*N.guibeiensis* sp. nov.	China: Guangxi: Xing’an: Maoershan (paratype)	NNU 00918	ON985181	ON968963	This study
3	*N.guibeiensis* sp. nov.	China: Guangxi: Xing’an: Maoershan (paratype)	NNU 00919	ON985182	ON968964	This study
4	*N.guibeiensis* sp. nov.	China: Guangxi: Xing’an: Yanguang (paratype)	NNU 00810	ON985179	ON968961	This study
5	*N.guibeiensis* sp. nov.	China: Guangxi: Xing’an: Zhongfeng (paratype)	NNU 00694	ON985176	ON968958	This study
6	*N.guibeiensis* sp. nov.	China: Guangxi: Xing’an: Zhongfeng (paratype)	NNU 00769	ON985177	ON968959	This study
7	*N.guibeiensis* sp. nov.	China: Guangxi: Xing’an: Zhongfeng (paratype)	NNU 00770	ON985178	ON968960	This study
8	*N.guibeiensis* sp. nov.	China: Guangxi: Xing’an: Zhongfeng (paratype)	NNU 00867	ON985183	ON968965	This study
9	*N.adenopleura*	China: Taiwan: Taichung City	SYS a007358	MN946445	MN945201	Lyu et al. (2020a)
10	*N.adenopleura*	China: Taiwan: Taichung City	SYS a007359	MN946446	MN945202	Lyu et al. (2020a)
11	*N.chapaensis*	Vietnam: Lao Cai: Sapa*	MNHN 2000.4850	KR827711	KR087625	Grosjean et al. (2015)
12	*N.daunchina*	China: Sichuan: Mt Emei*	SYS a004594	MF807822	MF807861	Lyu et al. (2020a)
13	*N.daunchina*	China: Sichuan: Mt Emei*	SYS a004595	MF807823	MF807862	Lyu et al. (2020a)
14	*N.guangdongensis*	China: Guangdong: Yingde City (holotype)	SYS a005767	MN946406	MN945162	Lyu et al. (2020a)
15	*N.guangdongensis*	China: Guangdong: Yingde City (paratype)	SYS a005768	MN946407	MN945163	Lyu et al. (2020a)
16	*N.guangxiensis*	China: Guangxi: Mt Daming (paratype)	NHMG 202007001	MZ677222	MZ678729	Lyu et al. (2021)
17	*N.guangxiensis*	China: Guangxi: Mt Daming (paratype)	NHMG 202007002	MZ677223	MZ678730	Lyu et al. (2021)
18	*N.hainanensis*	China: Hainan: Mt Diaoluo*	SYS a007669	MN946451	MN945207	Lyu et al. (2020a)
19	*N.hainanensis*	China: Hainan: Mt Diaoluo*	SYS a007670	MN946452	MN945208	Lyu et al. (2020a)
20	*N.leishanensis*	China: Guizhou: Mt Leigong*	SYS a007908	MN946453	MN945209	Lyu et al. (2021)
21	*N.leishanensis*	China: Guizhou: Mt Fanjing	SYS a007195	MN946454	MN945210	Lyu et al. (2021)
22	*N.leishanensis*	China: Guizhou: Mt Fanjing	SYS a007196	MN946455	MN945211	Lyu et al. (2021)
23	*N.leishanensis*	China: Guangxi: Mt Jiuwan	NHMG 202007021	MZ677227	MZ678734	Lyu et al. (2021)
24	*N.leishanensis*	China: Guangxi: Mt Jiuwan	NHMG 202007022	MZ677228	MZ678735	Lyu et al. (2021)
25	*N.leishanensis*	China: Guangxi: Mt Jiuwan	NHMG 202007023	MZ677229	MZ678736	Lyu et al. (2021)
26	*N.leishanensis*	China: Guangxi: Mt Jiuwan	NHMG 202007025	MZ677230	MZ678737	Lyu et al. (2021)
27	*N.lini*	China: Yunnan: Jiangcheng County*	SYS a003967	MF807818	MF807857	Lyu et al. (2017)
28	*N.lini*	China: Yunnan: Jiangcheng County*	SYS a003968	MF807819	MF807858	Lyu et al. (2017)
29	*N.mangveni*	China: Zhejiang: Mt Dapan (paratype)	SYS a006310	MN946424	MN945180	Lyu et al. (2020a)
30	*N.mangveni*	China: Zhejiang: Mt Dapan (paratype)	SYS a006311	MN946425	MN945181	Lyu et al. (2020a)
31	*N.nankunensis*	China: Guangdong: Mt Nankun (paratype)	SYS a005718	MF807839	MF807878	Lyu et al. (2017)
32	*N.nankunensis*	China: Guangdong: Mt Nankun (holotype)	SYS a005719	MF807840	MF807879	Lyu et al. (2017)
33	*N.occidentalis*	China: Yunnan: Mt Gaoligong (paratype)	SYS a003775	MF807816	MF807855	Lyu et al. (2020a)
34	*N.occidentalis*	China: Yunnan: Mt Gaoligong (holotype)	SYS a003776	MF807817	MF807856	Lyu et al. (2020a)
35	*N.okinavana*	Japan: Okinawa: Iriomote Island*	Unknown	NC022872	NC022872	Kakehashi et al. (2013)
36	*N.pleuraden*	China: Yunnan: Kunming City*	SYS a007858	MT935683	MT932858	Lyu et al. (2020b)
37	*N.pleuraden*	China: Yunnan: Wenshan City	SYS a007717	MT935671	MT932850	Lyu et al. (2020b)
38	*N.shiwandashanensis*	China: Guangxi: Shangsi County (holotype)	NNU00238	MZ787977	MZ782098	Chen et al. (2022)
39	*N.shiwandashanensis*	China: Guangxi: Shangsi County (paratype)	NNU00239	MZ787978	MZ782099	Chen et al. (2022)
40	*N.xiangica*	China: Hunan: Mt Dawei (paratype)	SYS a006491	MN946433	MN945189	Lyu et al. (2020a)
41	*N.xiangica*	China: Hunan: Mt Dawei (holotype)	SYS a006492	MN946434	MN945190	Lyu et al. (2020a)
42	*N.xiangica*	China: Hunan: Mt Dawei (paratype)	SYS a006493	MN946435	MN945191	Lyu et al. (2020a)
43	*N.xiangica*	China: Hunan: Mt Yangming (paratype)	SYS a007269	MN946436	MN945192	Lyu et al. (2020a)
44	*N.xiangica*	China: Hunan: Mt Yangming (paratype)	SYS a007270	MN946437	MN945193	Lyu et al. (2020a)
45	*N.xiangica*	China: Hunan: Mt Yangming (paratype)	SYS a007271	MN946438	MN945194	Lyu et al. (2020a)
46	*N.xiangica*	China: Hunan: Mt Yangming (paratype)	SYS a007272	MN946439	MN945195	Lyu et al. (2020a)
47	*N.xiangica*	China: Hunan: Mt Yangming (paratype)	SYS a007273	MN946440	MN945196	Lyu et al. (2020a)
48	*N.xiangica*	China: Jiangxi: Mt Wugong (paratype)	SYS a002590	MN946441	MN945197	Lyu et al. (2020a)
49	*N.xiangica*	China: Guangxi: Mt Dupangling	SYS a006568	MN946442	MN945198	Lyu et al. (2020a)
50	*N.xiangica*	China: Guangxi: Mt Dupangling	SYS a006569	MN946443	MN945199	Lyu et al. (2020a)
51	*N.xiangica*	China: Guangxi: Mt Dupangling	SYS a006570	MN946444	MN945200	Lyu et al. (2020a)
52	*N.yaoica*	China: Guangxi: Mt Dayao (paratype)	SYS a007020	MK882276	MK895041	Lyu et al. (2019)
53	*N.yaoica*	China: Guangxi: Mt Dayao (paratype)	SYS a007021	MK882277	MK895042	Lyu et al. (2019)
54	*N.yeae*	China: Guizhou: Tongzi County (paratype)	CIB TZ20190608005	MN295228	MN295234	Wei et al. (2020)
55	*N.yeae*	China: Guizhou: Tongzi County (paratype)	CIB TZ20160714016	MN295231	MN295237	Wei et al. (2020)
56	*Babinaholsti*	Japan: Okinawa*	Unknown	NC022870	NC022870	Kakehashi et al. (2013)
57	*Babinasubaspera*	Japan: Kagoshima: Amami Island*	Unknown	NC022871	NC022871	Kakehashi et al. (2013)

### ﻿Bioacoustics analysis

Advertisement calls of five individuals were recorded in the fields using a SONY ICX–0471 recorder on 7 May, and 3 and 28 June 2022. The ambient temperature was measured with a digital hygrothermograph. Calls were recorded at 21 °C, 23 °C, and 18 °C. Advertisement calls were analyzed using Raven Pro v. 1.6 (Cornell Laboratory of Ornithology, USA) as per Köhler et al. (2017). The acoustic properties were set to a window size of 512 points, fast Fourier transform, and Hanning window with no overlap. We performed the following measurements: call duration (measured from the beginning to the end of the call), note duration (measured from the beginning to the end of the note), inter-note interval (measured from the end of one note to the beginning of the consecutive note), and dominant frequency (the peak frequency of the call). The published bioacoustics data were obtained from the literature (Table 1).

## ﻿Results

**Figure 2.**
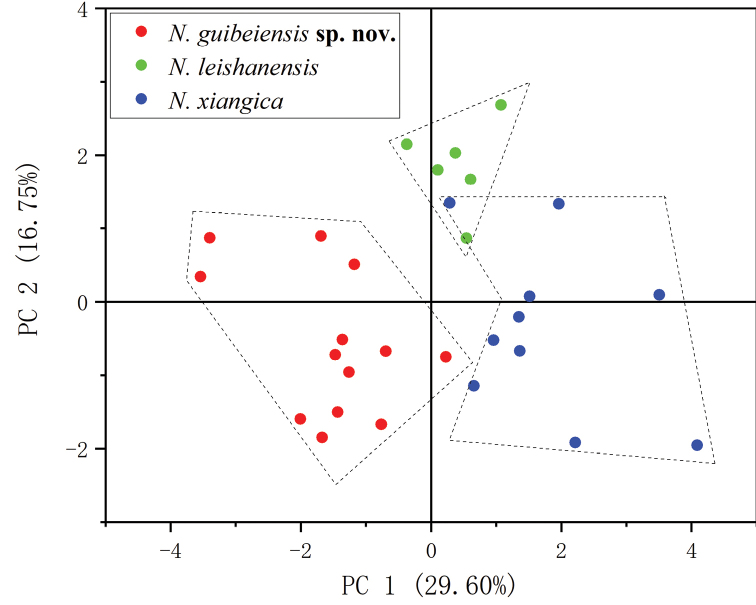
Scatter plot of PC1 and PC2 of PCA based on the morphometric measurements, distinguishing *Nidiranaguibeiensis* sp. nov., *N.leishanensis*, and *N.xiangica*.

**Figure 3.**
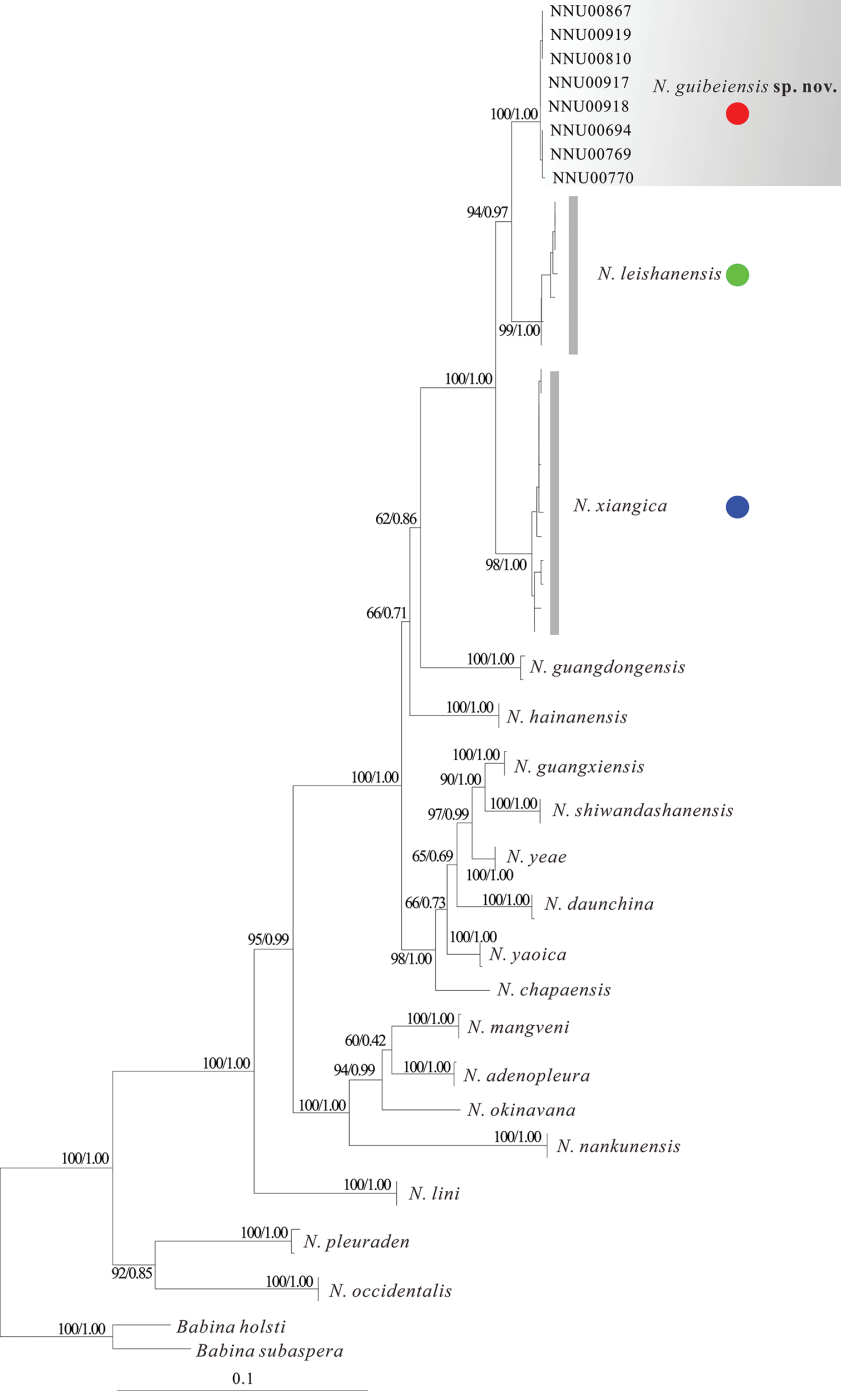
Maximum-likelihood tree based on 16S + COI fragments with bootstrap supports/Bayesian posterior probabilities on branches.

PCA results were shown in Fig. 2. The extracted components PC1 eigenvectors accounted for 29.6% of the variance, PC2 for 16.8%, and PC3 for 13.3%. The new specimens can be significantly distinguished from *N.leishanensis* and *N.xiangica*. The results of Mann-Whitney *U* tests implied that the new specimens were significantly different from *N.leishanensis* and *N.xiangica* on many morphometric characters, including HDW, SNT, IND, ED, and FTL for *N.leishanensis*, and SNT, IND, ED, HND, FTL, and TIB for *N.xiangica* (Table 3). Additionally, the new specimens can be easily identified by a series of diagnostic characters, such as relatively larger body size, smooth dorsum with tubercles on the posterior of the back, lateroventral grooves present on all fingers and toes but not on Finger I, and tibiotarsal articulation reaching the level between the eye and nostril (Table 1). ML and BI analyses led to identical topologies based on the two mtDNA fragments (Fig. 3). Phylogenetical trees indicated that our newly collected specimens were strongly clustered into a monophyletic group and sister to *N.leishanensis* with robust support (PP = 0.97, BS = 94).

**Table 3.** Measurements of *Nidiranaguibeiensis* sp. nov. (in mm) and morphometric comparisons with *N.leishanensis* and *N.xiangica*. Abbreviations defined in Material and methods. * *p*-values < 0.05, ** *p*-values < 0.01.

	Male (*n* = 13)		Female	*p*-values from Mann-Whitney *U* tests	
	Ranging	mean ± SD	NNU 00694	New species vs *N.leishanensis*	New species vs *N.xiangica*
SVL	50.2–63.6	55.2 ± 3.7	54.6	0.054	0.215
HDL	18.6–22.5	19.9 ± 1.1	19.8	0.380	0.137
HDW	18.3–23.1	20.1 ± 1.4	17.3	0.011*	0.321
SNT	7.0–9.5	7.5 ± 0.7	7.0	0.002**	0.003**
IND	5.3–6.6	6.0 ± 0.5	5.3	0.028*	0.000**
IOD	4.1–5.6	4.9 ± 0.4	5.0	0.661	0.121
ED	4.4–6.9	5.6 ± 0.7	5.4	0.028*	0.000**
TD	4.6–6.3	5.4 ± 0.5	4.8	0.161	0.094
HND	12.6–15.6	14.0 ± 1.0	14.3	0.726	0.041*
FTL	26.2–33.4	29.3 ± 2.0	30.5	0.001**	0.000**
TIB	25.7–29.9	28.4 ± 1.5	30.8	0.861	0.001**

**Figure 4.**
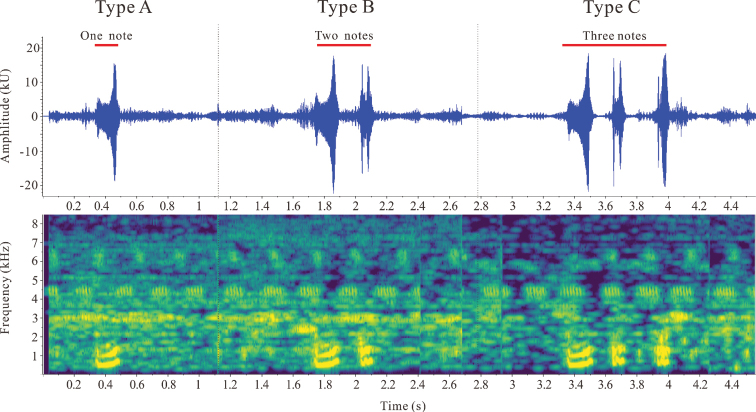
Advertisement call spectrograms of *Nidiranaguibeiensis* sp. nov.

The call spectrograms of the new specimens are shown in Fig. 4. Three types of calls were recorded: calls with one note (Type A), two notes (Type B), and three notes (Type C). Note number of a call, call duration, note duration, and inter-note duration are listed in Table 4. The newly collected specimens had different call characteristics distinguishing them from the call types of their congeners, a call with notes, call duration, and note duration (Table 4). However, the calls of *N.leishanensis* consist of one strophe with one syllable, and call durations last 330–430 ms. The calls of *N.xiangica* consist of two or three notes, and call durations last 331.9–624.8 ms. The two species above are distinct from the new specimens based on acoustic data (Table 4).

**Table 4.** Vocalization parameters of *Nidiranaguibeiensis* sp. nov.

	Type A (*n* = 21)	Type B (*n* = 37)	Type C (*n* = 13)
Notes number of one call	1	2	3
Call duration	155–232, (mean 179.6 ± 24.4) ms	349–471, (mean 383.4 ± 28.6) ms	561–777, (mean 658.6 ± 71.7) ms
First note duration		153–210, (mean 167.4 ± 13.1) ms	160–210, (mean 187.8 ± 18.4) ms
Second note duration		71–90, (mean 79.3 ± 4.2) ms	71–86, (mean 77.2 ± 5.5) ms
Third note duration			75–93, (mean 82 ± 5.3) ms
First inter-note duration		125–171, (mean 136.6 ± 11.3) ms	113–167, (mean 139.5 ± 18.3) ms
Second inter-note duration			142–221, (mean 171.8 ± 24.1) ms

Phylogeny, bioacoustics, and morphology support the recognition of the newly collected specimens from northern Guangxi as a previously undescribed *Nidirana* species, which is described below.

### ﻿Taxonomic account

#### ﻿ Nidirana guibeiensis

Taxon classificationAnimaliaAnuraRanidae

Chen, Ye, Peng & Li sp. nov.

71C60F29-AF0C-5124-9E31-22A1C3DB122C

https://zoobank.org/22E61BF0-A1D2-4E44-83A5-F4EA99A50B78

Fig. 5

##### Holotype.

NNU 00771; adult ♂; China, Guangxi, Ziyuan County, Zhongfeng Town; 110.6882°E, 25.9750°N; Wei-Cai Chen leg., 8 May 2022.

##### Paratypes.

NNU 00769–770, 772–773; 4 adult ♂♂; same locality and date as holotype • NNU 00694; 1 adult ♀; same locality as holotype; Gui-Dong Yu leg., 29 April 2022 • NNU 00810; 1 adult ♂; China, Guangxi, Xing’an County, Lingtan Town; 110.5622°E, 25.5907°N; Wei-Cai Chen leg., 9 May 2022 • NNU 00864–867; 4 adult ♂♂; same locality as holotype; Wei-Cai Chen leg., 2 June 2022 • NNU 00917–919; 3 adult ♂♂; Maoershan National Nature Reserve; 110.4937°E, 25.8823°N; Wei-Cai Chen, Tong-Ping Su & Gui-Dong Yu leg., 28 June 2022.

##### Etymology.

The species name refers to its distribution in northern Guangxi. ‘Guibei’ means northern Guangxi.

We suggest the English name Guibei Music Frog and the Chinese name Gui Bei Qin Wa (桂北琴蛙).

##### Diagnosis.

*Nidiranaguibeiensis* sp. nov. differs from its congeners in the combination of the following characteristics: larger body size (SVL 50.2–63.6 mm in males; 54.6 mm in the only sampled female); dorsum smooth with tubercles on the posterior of the back; surfaces of throat, chest, and upper part of the belly with grey clouding, lower part of the belly near immaculate creamy white; dorsal midline with a discrete creamy-white line; lateroventral grooves present on all fingers and toes but not on Finger I; tibiotarsal articulation reaching the level between eye and nostril; a pair of subgular vocal sacs present; three types of calls: one note, two notes, or three notes.

##### Description of holotype.

**Figure 5.**
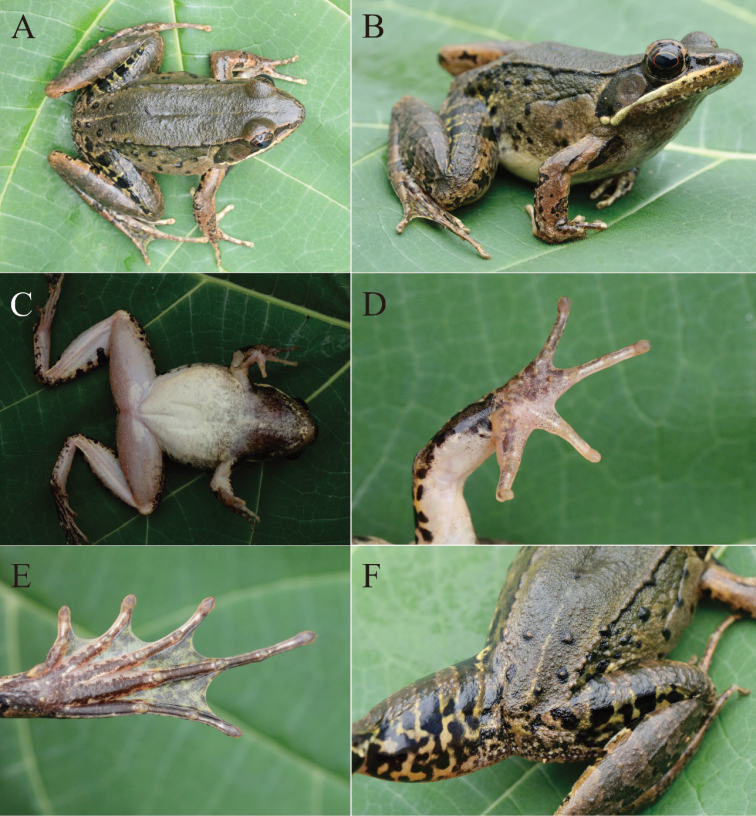
The holotype of *Nidiranaguibeiensis* sp. nov. (NNU 00771) **A** dorsal view **B** dorsolateral view **C** ventral view **D** ventral view of hand **E** ventral view of foot **F** tubercles on the rear of the back.

Adult male, SVL 56.3 mm; head length slightly larger than width (HDL/HDW = 1.05); snout oval, significantly protruding beyond lower jaw; canthus rostralis distinct; loreal region concave; nostril oval and closer to snout than eye, laterally opening; a creamy white stripe on upper lip, beginning at the tip of snout along with upper lip and ending above insertion of arm; supratympanic fold visible; IOD/IND = 0.72; eye diameter almost equal to tympanum diameter (ED/TD = 1.02); vomerine teeth oval, closer to each other than to choana; tongue pyriform with a deep notch on posterior; a pair of subgular vocal sacs present. Relative finger lengths: II < IV < I < III; all tips of fingers but Finger I slightly dilated with lateroventral grooves; finger webbing and dermal fringes absent; subarticular tubercles prominent and conical; two palmar tubercles distinct; nuptial pad present on lateral Finger I with velvety spinules, extending from hand base to level of subarticular tubercle. Relative toe lengths: I < II < V < III < IV; all tips of toes slightly dilated, forming elongated and pointed discs with lateroventral grooves; toe webbing formula: I 2 – 2 II 1½ – 3– III 2 – 3^+^ IV 3^+^ – 2– V; toes with lateral fringes; subarticular tubercles prominent and oval; inner metatarsal tubercles elongated, but outer metatarsal tubercles conical; heels not meeting when thighs are held at right angles to body; tibiotarsal articulation reaching the level between eye and nostril. Dorsum smooth with tubercles on the posterior of the back; hindlimbs smooth with several tubercles; dorsolateral fold beginning at the posterior of eye and ending above groin; pineal gland distinct; flanks with suprabrachial glands at each side; peripheral vent with some small tubercles (Fig. 5A–F).

##### Color of holotype.

Alive, dorsum moss grey without spots; pineal gland light yellow; dorsal midline with a discrete creamy-white line, beginning at pineal gland and ending at vent; tympanum light brown; presence of a creamy-white linear gland on upper jaw; maxillary gland creamy-white; flank with several black spots and tubercles, and a large grey suprabrachial gland; thigh and tibia with three distinct black bars; surfaces of throat, chest, and upper part of belly with grey clouding, lower part of belly near immaculate creamy white; ventral limbs incarnadine; anterior of base of forelimb with a dark stripe; the anterior and posterior of iris reddish-brown, whereas the upper and lower part of iris brown (Fig. 5A–F). In preservation, dorsal surface faded to deep grey; black spots turned darkish black.

##### Variations.

**Figure 6.**
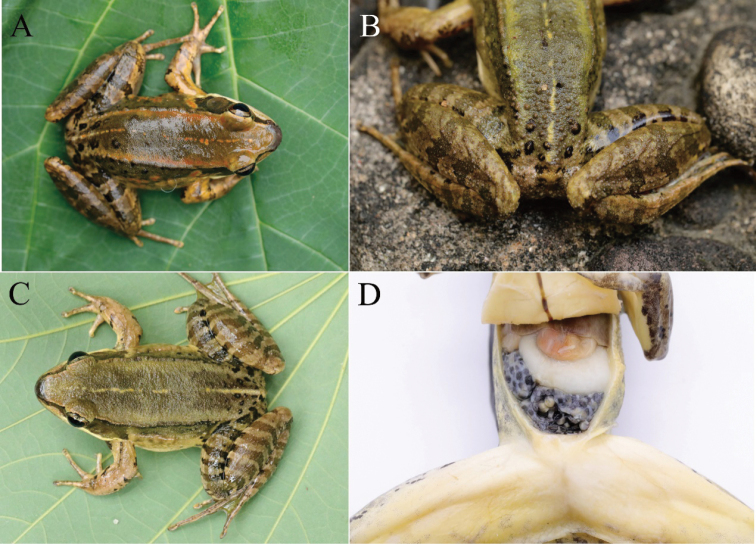
*Nidiranaguibeiensis* sp. nov. **A** dorsal view of NNU 00769 **B** rough tubercles on the rear of the back (NNU 00865) **C** five bars on thigh and tibia (NNU 00867) **D** female with creamy yellow eggs with pigmented poles (NNU 00694).

Measurements of type series are listed in Table 3 and Table S1. Paratypes were similar to the holotype in morphology and color pattern. Some had a discrete, rusty dorsal midline, and a rusty line along the dorsolateral folds, and rusty blotches on the flanks (Fig. 6A). Various tubercles on the rear of the back, some denser but some sparse (Fig. 6B). Some had five or six black bars on thigh and tibia (Fig. 6C).

##### Ecology and distribution.

**Figure 7.**
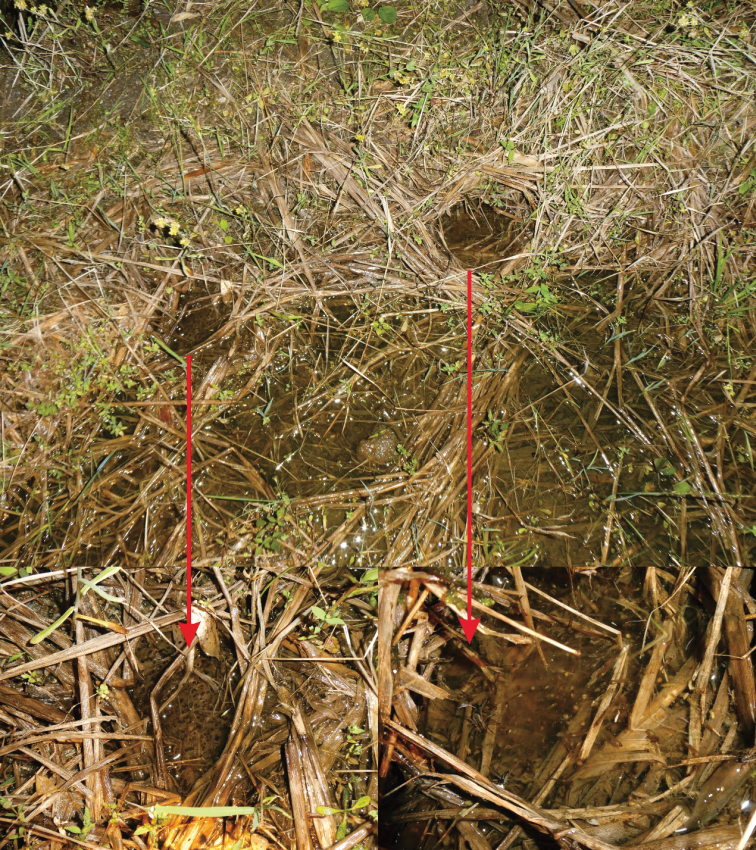
Nests of *Nidiranaguibeiensis* sp. nov. with eggs.

*Nidiranaguibeiensis* sp. nov. was found in paddy fields or still pools at 300–1 300 m a.s.l. We heard the advertisement calls in the field during the surveys, from April to July. We observed that the new species has nest construction behavior (Fig. 7). The nest was made of rice stems and 20–30 cm in diameter without a covering. Eggs were observed in the nest (Fig. 7). Females were gravid with creamy-yellow eggs with black poles (Fig. 6D). The new species is widespread in northern Guangxi.

##### Comparison.

A summary of morphological characteristics is listed in Table 1. *Nidiranaguibeiensis* sp. nov. differs from its congeners in the following characteristics: (1) SVL 50.2–63.6 mm in males; (2) dorsum smooth with tubercles on the posterior of the back; (3) surfaces of throat, chest, and upper part of belly with grey clouding, lower part of belly near immaculate creamy white; (4) dorsal midline with a discrete creamy-white line; (5) tibiotarsal articulation reaching the level between eye and nostril; (6) lateroventral grooves present on all fingers and toes but not on Finger I; (7) nuptial pad present on Finger I; (8) a pair of subgular vocal sacs present; (9) a call comprised of one, two, or three notes.

Phylogenetically, *N.guibeiensis* sp. nov. is closest to *N.leishanensis* (Fig. 3). However, *N.guibeiensis* sp. nov. differs from *N.leishanensis* in the absence of dermal fringes on fingers (vs broad lateral fringes on inner sides of Fingers II, III, and IV but absent on Finger I); the presence of lateroventral grooves on all fingers except Finger I (vs lateroventral grooves present on Fingers III and IV); toe webbing formula: I 2 – 2 II 1½ – 3– III 2 – 3^+^ IV 3^+^ – 2– V (vs I 1⅓ –2 II 1⅓ –2⅓ III 1⅔ –3 IV 3⅓ –1⅓ V); dorsum smooth with tubercles on the posterior of the back (vs dorsal skin rough with dense granules but not concentrated on the posterior of the back); heels not meeting when thighs are held at right angles to body (vs heels overlapping); outer metatarsal tubercle present (vs absent); surfaces of throat, chest, and upper part of belly with grey clouding, lower part of belly near immaculate creamy white (vs surface and throat smooth and incarnadine); supratympanic fold present (vs absent); two palmar tubercles distinct (vs three palmar tubercles elliptic, distinct); nuptial pad present on Finger I (vs nuptial pad on the inner side of base of Fingers I and II); a call comprised of one, two, or three notes (vs a call with one strophe with one syllable). The new species has nest construction behavior (vs no nest construction behavior).

*Nidiranaguibeiensis* sp. nov. differs from *N.xiangica* in having a dorsal midline with a discrete creamy-white line (vs absent dorsal midline); heels not meeting when thighs are held at right angles to body (vs heels meeting); a smooth dorsum with tubercles on the posterior of the back (vs extremely rough dorsal surface with dense tubercles and white horny spinules on the entire dorsum); supratympanic fold present (vs absent); the anterior and posterior of iris reddish-brown, whereas the upper and lower parts of iris brown (vs upper ⅓ iris brownish-white and lower ⅔ iris reddish-brown); advertisement calls contained 1–3 notes (vs 2 or 3 notes with a specific first note). Additionally, the new species has nest construction behavior (vs no nest construction behavior).

Due to the larger body size in males (SVL 50.2–63.6 mm), *N.guibeiensis* sp. nov. differs from males of *N.nankunensis* (SVL 33.3–37.1 mm), *N.chapaensis* (SVL 35.5–42.5 mm), *N.guangxiensis* (SVL 40.2–47.6 mm), *N.hainanensis* (SVL 32.8–44.4 mm), *N.okinavana* (SVL 35.5–42.8 mm), *N.yaoica* (SVL 42.1–45.6 mm), and *N.yeae* (SVL 41.2–43.5 mm). Other differences are (Table 1): lateroventral grooves present on all fingers but not on Finger I (vs absent in *N.guangxiensis*, *N.occidentalis*, *N.pleuraden*, and *N.yeae*); lateroventral grooves present on all toes (vs absent on all toes in *N.pleuraden* and *N.occidentalis*) ; tibiotarsal articulation reaching the level between the eye and nostril (vs beyond the tip of snout in *N.lini*); dorsum smooth with tubercles on the posterior of the back (vs no tubercles on the posterior of the back in *N.chapaensis*, *N.daunchina*, *N.guangdongensis*, *N.guangxiensis*, *N.hainanensis*, *N.leishanensis*, *N.nankunensis*, *N.okinavana*, *N.shiwandashanensis*, *N.yaoica*, and *N.yeae*); single nuptial pad present on Finger I (vs absent in *N.hainanensis*; two parts on Finger I in *N.chapaensis*; present on Fingers I and II in *N.leishanensis*).

## ﻿Discussion

To date, six recognized *Nidirana* species have been reported from Guangxi, indicating an impressive species diversity. Lyu et al. (2021) and Chen et al. (2022) pointed out that *Nidirana* species have relatively narrow ranges. Rivers and mountains contribute to the speciation of *Nidirana*. Phylogenetically, the new species, *N.leishanensis*, and *N.xiangica* are clustered together. The three species are geographically close but occur in different mountain ranges (Fig. 1). *Nidiranaguibeiensis* sp. nov. occurs in the Yuechengling Mountains, *N.leishanensis* in the Miaoling Mountains, and *N.xiangica* in the Dupanling, Dawei, and Yangming mountains. The other three species of *Nidirana* occurring in Guangxi (*N.guangxiensis*, *N.shiwandashanensis* and *N.yaoica*) resemble the abovementioned species but occur in separated mountain ranges (Lyu et al. 2019, 2021; Chen et al. 2022).

The discovery of the new species indicates that the diversity of the genus *Nidirana* is still underestimated. The taxonomic validity of reports of *N.adenopleura* in Guangxi must be reconsidered. In recent years, we have carried out a series of field surveys, but no evidence supports the occurrence of *N.adenopleura* in Guangxi. *Nidiranaguibeiensis* sp. nov., which was reported by Mo et al. (2014) as *N.adenopleura*, is a good example of a misidentification of a new *Nidirana* species.

## Supplementary Material

XML Treatment for Nidirana guibeiensis

## ﻿Appendix 1

**Table A1.** Specimens examined. Type locality indicated by an asterisk (*).

Species	Locality	Voucher no.
*Nidiranaguangxiensis*	Damingshan National Nature Reserve, Guangxi, China*	NNU 00213
*Nidiranaguangxiensis*	Damingshan National Nature Reserve, Guangxi, China*	NNU 00214
*Nidiranaguangxiensis*	Damingshan National Nature Reserve, Guangxi, China*	NNU 00215
*Nidiranaguangxiensis*	Damingshan National Nature Reserve, Guangxi, China*	NNU 00216
*Nidiranaguangxiensis*	Damingshan National Nature Reserve, Guangxi, China*	NNU 00217
*Nidiranaguangxiensis*	Damingshan National Nature Reserve, Guangxi, China*	NNU 00218
*Nidiranaleishanensis*	Yuanbaoshan National Nature Reserve, Guangxi, China	NNU 201908032
*Nidiranaleishanensis*	Yuanbaoshan National Nature Reserve, Guangxi, China	NNU 201908033
*Nidiranashiwandashanensis*	Shiwandashan National Nature Reserve, Guangxi, China*	NNU 00238
*Nidiranashiwandashanensis*	Shiwandashan National Nature Reserve, Guangxi, China*	NNU 00239
*Nidiranashiwandashanensis*	Shiwandashan National Nature Reserve, Guangxi, China*	NNU 00605
*Nidiranashiwandashanensis*	Shiwandashan National Nature Reserve, Guangxi, China*	NNU 00606
*Nidiranayaoica*	Dayaoshan National Nature Reserve, Guangxi, China*	NNU 201907008
*Nidiranayaoica*	Dayaoshan National Nature Reserve, Guangxi, China*	NNU 201907009

## Supplementary materials

Supplementary material 1 Supplementary data Data type Morphological. Explanation note Measurements of *Nidiranaguibeiensis* sp. nov. (in mm). Abbreviations defined in Material and methods. *= holotype. https://binary.pensoft.net/file/781157This dataset is made available under the Open Database License (http://opendatacommons.org/licenses/odbl/1.0/). The Open Database License (ODbL) is a license agreement intended to allow users to freely share, modify, and use this Dataset while maintaining this same freedom for others, provided that the original source and author(s) are credited.Wei-Cai Chen, Jian-Ping Ye, Wan-Xiao Peng, Peng Li, Tong-Ping Su, Gui-Dong Yu, Zhi-Ying Cheng
