# Supplementary material for: ﻿A new species of Nidirana (Anura, Ranidae) from northern Guangxi, China
Source: Zookeys. 2022 Dec 12;1135:119–37. doi: 10.3897/zookeys.1135.94371 (PMC9836524; doi:10.3897/zookeys.1135.94371)
Supplement: Supplementary material 1 — Supplementary data [file zookeys-1135-119_article-94371__-s001.docx]

**Table S1** Measurements of *Nidirana guibeiensis* **sp. nov.** (in mm). Abbreviations defined in Material and methods. * = holotype.

| Voucher | Sex | SVL | HDL | HDW | SNT | IND | IOD | ED | TD | HND | FTL | TIB |
| --- | --- | --- | --- | --- | --- | --- | --- | --- | --- | --- | --- | --- |
| NNU 00769 | Male | 59.8 | 20.4 | 21.4 | 7.4 | 6.6 | 5.1 | 5.7 | 5.5 | 14.7 | 29.5 | 28.3 |
| NNU 00770 | Male | 55.1 | 18.6 | 20.7 | 7.5 | 6.2 | 4.6 | 5.8 | 5.6 | 14.0 | 28.7 | 28.4 |
| NNU 00771* | Male | 56.3 | 20.1 | 19.2 | 7.5 | 6.4 | 4.6 | 5.4 | 5.3 | 14.4 | 29.9 | 29.6 |
| NNU 00772 | Male | 51.9 | 18.8 | 18.3 | 7.2 | 5.6 | 4.1 | 4.4 | 5.1 | 13.4 | 27.9 | 27.1 |
| NNU 00773 | Male | 50.2 | 18.9 | 18.4 | 7.0 | 5.3 | 4.8 | 4.4 | 5.2 | 12.8 | 26.3 | 25.7 |
| NNU 00810 | Male | 57.9 | 19.5 | 19.9 | 8.0 | 6.6 | 5.3 | 5.8 | 6.1 | 15.3 | 30.5 | 29.7 |
| NNU 00864 | Male | 52.0 | 19.6 | 18.8 | 7.6 | 5.7 | 4.5 | 5.6 | 5.3 | 12.7 | 26.2 | 26.1 |
| NNU 00865 | Male | 54.6 | 21.2 | 20.8 | 7.5 | 5.7 | 5.2 | 5.6 | 5.5 | 13.8 | 29.6 | 28.4 |
| NNU 00866 | Male | 52.9 | 19.6 | 19.5 | 7.0 | 5.7 | 4.6 | 5.6 | 5.3 | 12.6 | 29.1 | 28.9 |
| NNU 00867 | Male | 53.2 | 19.3 | 20.6 | 7.3 | 5.9 | 5.0 | 5.4 | 5.4 | 13.8 | 29.7 | 28.9 |
| NNU 00917 | Male | 63.6 | 22.5 | 23.1 | 9.5 | 6.4 | 5.6 | 6.9 | 6.3 | 15.6 | 33.4 | 29.2 |
| NNU 00918 | Male | 55.6 | 21.0 | 20.4 | 7.1 | 6.4 | 5.0 | 6.0 | 4.9 | 15.0 | 30.6 | 29.9 |
| NNU 00919 | Male | 54.1 | 19.6 | 20.1 | 7.5 | 5.3 | 5.2 | 6.2 | 4.6 | 14.1 | 29.8 | 28.9 |
| NNU 00694 | Female | 54.6 | 19.8 | 17.3 | 7.0 | 5.3 | 5.0 | 5.4 | 4.8 | 14.3 | 30.5 | 30.8 |
| NNU 00774 | Subadult | 31.0 | 12.9 | 11.0 | 5.3 | 3.7 | 3.3 | 2.8 | 2.8 | 8.7 | 17.7 | 16.7 |
